# Books Reviewed

**Published:** 1868-01

**Authors:** 


### Editorial Department.

### Books Reviewed.

A Contribution to the History of the Hip-joint Operations performed during the late Civil War in the Confederate service. By Paul F. Eve, M. D., Professor of Surgery in the University of Nashville, Tenn.

This pamphlet presents the statistics of twenty amputations and thirteen resections at the coxo-femoral articulation occurring in the Confederate service during the late war. Of the twenty amputations three primary and one secondary operation proved successful, giving a ratio of one in five. This result Prof. Eve rather exultingly compares with that presented in Circular No. 6, War Department, in which twenty-one hip-joint amputations are recorded, three of which proved successful. One of these cases appears from later investigations to have been erroneously recorded, thus reducing the ratio of mortality to one in ten, which would make it double to that of the Southern service. We greatly regret that one of the most distinguished and able surgeons of the South, and one whose statements are so universally accepted, should have permitted himself to arrive at such premature conclusions, for since the publication of the source from which Professor Eve has drawn his information, and while he had his pamphlet in construction, the War Department was preparing and shortly afterwards published Circular No. 7, July 1, 1867, a Report on the Amputations at the Hip-joint. From this report it appears that thirty-four coxo-femoral disarticulations were performed by Federal surgeons, one recovering after primary, two after secondary, and four after re-amputations, giving a total of seven recoveries, or a little more than one in five.

In reviewing the cases claimed by Professor Eve as successful, we first notice the case of Dr. Grant, reported as a successful primary amputation. This patient was operated upon October 19, 1862; "he was taken to a neighboring house where he was seen by the operator three days afterwards. The Federal forces occupying the place Dr. G. did not see his patient again, but heard from him October 30, eleven days afterwards, but no information was ever after received, and it is altogether probable that the patient died," as stated in the Surgeon-General's Report. Again, the case of private Robinson, upon whom a primary amputation was performed by Surgeon Compton, cannot be regarded as authenticated beyond doubt, since the history of this case is not traced beyond six months after the operation, at which time the patient reported himself in "good condition." This case is excluded also by the Surgeon-General.

Furthermore, we direct attention to the discrepancy of statement, that these results were obtained under the most unfavorable circumstances due to "the destitute and isolated condition of the South." Although. in itself, this state ment may be perfectly correct, yet it does not appear that this class of patients did not receive all the sanitary comforts which they required, Dr. A. T. Gilman feeling "convinced" that the recovery of Williamson was due to the nutritious regimen upon which he was placed, "a messenger being sent daily to Richmond for egg, milk, and other delicacies." Nor does it appear that *any* of this class of patients suffered more hygienically than those of their Federal brethren, while all were more favorably situated as regards the climatic influences.

In table No. 2, thirteen cases of resection of the head of the femur are tabulated, four of which were successful, or one in every two and a half cases. In Circular No. 6, War Department, S. G. O., thirty-two cases are recorded, with but four recoveries, or a ratio of one to eight. While we are disposed to give all due credit to the successes of our Southern friends, we are fully persuaded that when the history of this class of operations will have been as carefully studied and recorded as that of hip-joint amputations, no such disparaging difference will be found to exist as the investigation of Professor Eve would make it appear.

---

Shock, after Surgical Operations and Injuries, with special reference to shock caused by Railroad Accidents. By Edwin Morris, M. D., F. R. C. S. Philadelphia: J. B. Lippincott & Co.

Upon the meaning and true nature of "Shock" a great deal has been written, and authors have endeavored faithfully to explain its causes and modes of operation. The author of this work has added to our literature upon the subject a very carefully considered discussion of all the facts and circumstances attending the condition known as *shock*, and every one must be deeply interested in careful perusal of the volume. We indulge in an extract to show what the author has collected upon the generally believed erroneous statement, that hair may turn white, in a single night, from mental emotion.

"Shock from mental causes has a very powerful effect upon the human frame. 'A lady who was deeply *shocked* on receiving the intelligence of a great change in her worldly condition, and who had a remarkable quantity of dark hair, found on the following morning that the whole of her hair had become of a silver white.'

'My hair turned white
In a single night,
Like some have done
With sudden fears.'—BYRON.

Several historical writers have recorded instances of the severity of mental *shock*. 'I was struck,' says Madame Campau, 'with the astonishing change misfortune had brought upon Marie Antoinette's features; even her hair had turned almost *white* during her transit from Verennes to Paris.' 'The Duchess of Luxembourg was caught making her escape during the horrors of the French Revolution, and put in prison; the next morning it was observed that her hair had become *white*.'

'A practical joke was played upon a brave Spanish officer of the Duke of Alva's camp, to try his courage. The provost marshal, with a guard and a confessor, awoke him in the night from his sleep, stating that he had an order for his immediate execution. He confessed, said he was prepared to die, but declared his innocence. The provost marshal laughed, and said it was only a joke. With a ghastly paleness, he ordered the provost out of the tent, saying that he had 'done him an evil office,' and the next morning, to the wonder of the whole army, the hair of his head, from a deep *black* color, had become perfectly *white*.'

Wright on Headaches. Philadelphia: Lindsay & Blakiston, 1867.

This is a little book discussing, quite philosophically, all the causes of the various affections classed as "headaches." He has almost regarded and treated headache as a disease, appearing under widely differing circumstances, and from a great many different causes, though we do not think he has any such view of it, or holds to any other than the most correct opinions. The relation headache bears to disease as a symptom, and the importance of not neglecting it under most circumstances, together with the best means of cure, seems to be the topics upon which the book is written. The practical question of treatment is evidently uppermost, and to it has been devoted a great amount of attention. The remedies proposed are those well known to the profession, but a full table of Formulæ which is appended to the work will be attractive to those who desire to perfect themselves in the old English method of combining nearly all compatible and valuable medicines in the same mixture.

His discussion of disease and its causes, is very rational and consistent, and his methods of cure are not greatly objectionable, the only fault, so far as we observe, being the administration of medicine in too great variety. This work will be found very interesting and instructive upon a symptom of disease, worthy of more careful attention than it usually receives.

---

Lectures on the Diseases of Women. By Charles West, M. D. Philadelphia: Henry C. Lea.

The well known standing and merit of this work makes it quite unnecessary for us to go into any detailed statement of the doctrines it contains, or the subjects upon which it treats. It has long been regarded as one of the standard books upon this department of medicine, and includes a consideration of nearly every topic which comes within its scope. The chief changes in this edition will be found under the heads of uterine hæmatocele and ovarian diseases, though the whole work has been carefully revised and additions made whenever increased knowledge afforded the possibility of making important improvements. We have long regarded West on the Diseases of Women, as really one of the best works upon the subject; correct, complete, candid, practical, and every way worthy of confidence.

---

The Atlantic Monthly.

This valuable journal, devoted to literature, science and art, is, beyond all doubt, the most attractive, interesting and popular journal of its kind published in this country, being a never-failing source of instruction and interest.

Every Saturday.

This periodical aims to give its readers the best and most readable papers that appear in European Magazines and Reviews, and is almost indispensable to every intelligent American. Ticknor & Fields, Boston, are the publishers of the above journals.

Stillé on Epidemic Meningitis.

This is a beautiful, complete, and scientific treatise upon Epidemic Meningiti s a disease which, until its morbid anatomy was discovered and carefully studied, had passed under various and vague names, all, now showing the absolute ignorance prevailing as to its true nature. The local lesion appears to have been discovered many years since, but the relation which the local disease held to the general phenomena must have been unappreciated. We should like to make copious extracts from the work, but every physician should be in possession of the book, which contains too much fact and conclusion to be neglected by any one who means to keep himself acquainted with the progress of practical medicine.

---

Carson's Synopsis of Materia Medica.

This outline of Materia Medica has been prepared by Prof. Carson, for the special benefit of students attending his Lectures, in the University of Pennsylvania. It is only a frame work, which the author says, may be, with ordinary in, dustry, filled in, by notes, taken at the time the lectures are delivered. Students attending the Lectures of the author, will certainly appreciate the work; students elsewhere might make it useful, but probably not to such degree as those attending his course.

---

A Treatise on the Causes of exhausted Vitality: or Abuse of the Sexual Functions. By E. P. Miller, M. D. New York: 1867.

The prejudices and repugnance pervading the public against any discussion of the sexual functions has generally deterred physicians from publicly directing attention to the evils arising from the abuse of these functions until silence is no longer a virtue. While Prof. H. R. Storer, in his excellent monographs, has pointed out the dangers arising from procured abortion, etc., Dr. Miller calls attention to the dangerous practices so extensively and ruinously pervading among the young and unmarried, The author has presented the subject in all its relations, in a plain and practical manner, and we have no doubt that this book will fulfill its mission wherever read.

